# The Future of the Hospitals

**Published:** 1910-08-06

**Authors:** 


### THE FUTURE OF THE HOSPITALS.

### THE BRITISH MEDICAL ASSOCIATION'S DISCUSSION ON THE ECONOMIC BASIS OF HOSPITAL MANAGEMENT.

At the meeting of the Medico-Sociological Section, held at the College of Science, South Kensington, on Wednesday, July 27, 1910, Dr. James A. Macdonald (Taunton) in the chair, the chairman, in opening the meeting, said the small attendance was due to the fact that the State Medicine Section was holding its sittings at the same time. The latter had been spoken of three years ago, but the decision to open the Medico-Sociological Section was only come to last year at Belfast, and considerations of medical practice in Ireland were so different from those in England that the present was the first meeting of it. Some might think it strange that there should be such a section in the British Medical Association, but the Association existed for the furtherance of medicine and the allied sciences, and sociology was certainly allied to medicine. The conditions of life had altered so much in the last few years, since the State had taken an increased interest in the public, that there were many different ways of dealing with medicine in relation to the State and the individual. Since 1835 it had been the pleasure of people at large to consider that the medical profession was one which was made for the benefit of being exploited by other people, an attitude which the profession itself had largely contributed to. But members of that profession had now decided that it was time they looked after their own affairs, as the claims made for their services without fee were increasingly numerous. The first work of the section was to lay down the lines on which sociology was to be dealt with. It was necessary to avoid trenching on the domain of party politics, and the scope was wide enough without doing so.

### Professor Moore on the Voluntary System.

Professor Moore, in opening the debate on "The Economic Basis of Hospital Management," said the subject was of the widest possible scope, and would serve to indicate the present position of medical sociology. On account of the many changes which were now occurring in the way in which medical practice was carried out in this country, there had come to be a strong feeling of aversion on the part of the profession towards the method in which our hospitals were conducted. But changes had been introduced by the Government which seemed to be capable, if they were handled aright, of doing away with those objections, and of bringing our hospital work and the work of the medical practitioner into line to a greater extent than had yet been attained. It was necessary to consider what was wrong in the present hospital organisation; in what way was it out of touch with the wants of the profession, and in what way, if any, did the hospital ill-treat the profession? Most of the London medical schools began in the reign of Henry VIII., because there was no way of providing with treatment very poor people who were afflicted with disease. They had been in the habit of lying about afflicted with all kinds of loathsome disease. The keynote of hospitals was benevolence for the individual case. And the Church of to-day led the way. The monks were the physicians to these hospitals, they formed the "spitals," into which the poor sick were taken. As the science of medicine progressed hospitals had altered, but the relationship of the medical school to the hospital had largely persisted to the present time. It was necessary now to take a different point of view of the hospital altogether than that it was a charitable institution for the good of the individual. While it did good for the individual it also went further. The first function of the hospital was the study of disease and advance in the methods of study, which could only take place under a co-ordinated system. Secondly, there was the training of the future medical practitioners. No one would think of going back to the old apprenticeship system, under which a man learning his business as a doctor was trained under one man alone. It had also to be remembered that in the hospitals the poor class were treated, and the ultimate physical wellbeing of the nation was dependent on the way in which that class was treated. One type of organism for a particular disease affected the rich man and the poor. Therefore hospitals were good not merely from the stand point of pity or charity, but they should be supported for their own sake and for the sake of the nation as a whole.

### Municipalisation.

In reality the hospital was a national institution. What were the disadvantages produced by the charitable aspect of dealing with hospitals? The funds at the disposal of the hospital became insufficient because a large number of people were not charitable. If nine out of ten wealthy men did not feel disposed to contribute, no compulsion could be placed upon them, and the burden was thus unevenly distributed. Thus for the protection of the nation it was necessary that municipalities and public means should be used. Public authorities when endowing a hospital always had in mind that they were appointed to keep the rates down; hence those hospitals were often inadequately equipped for their work. He had seen many institutions, and he wished to declare that any institution which expected one resident medical man to look after more than 500 beds was a disgrace to the age in which we live. In many cases we were as badly off to-day with regard to the Poor Law hospitals as were the prisons in the days of John Howard. He had been in a large hospital of the kind where phthisical and other kinds of patients were treated in a large room which had no windows on three sides of it. There was no co-ordination at all. Surely that was not in accordance with modern science.

### Some Grievances.

Patients suffering from infectious diseases were discharged from hospital straight back to mix with the community. Every case of infectious disease must, before it died, infect somebody else, and hence that discharge of patients was a real danger. Another important point in connection with hospitals on the voluntary system was that the governing body consisted largely of laymen, not medical men, and when an appointment became vacant there was all the canvassing which was so objectionable, and the determining factor was more likely to be the number of acquaintances which the applicant had on the Board than his medical or surgical ability. There was also a want of co-ordination between the resident posts and the senior posts. In every other national service the man best fitted for a post by ability was chosen to fill it, and there were means and avenues open for his promotion. A man once appointed to a hospital might be tied to that one for life. Though it sometimes happened, it was seldom that a man in such a post moved from one city to another. That bar should be got rid of, and it should be possible to choose a man from any part of the country. The Poor Law hospitals should certainly be brought into co-ordination. A medical man took up a Poor Law appointment not because he wanted to be a member of that service, but because he wanted six months' work and experience of that kind, and perhaps required the D.P.H. The bulk of the men who entered that service left it later and went in for something else. There was no promotion in the Poor Law service until one got to a certain grade, and one got an appointment often because members of the lay governing body knew the applicant.

Hospital Abuse.

There were many ways of entrance into hospitals on the part of patients. A sore point was the admission into hospitals of people who were quite able to pay. Salvation in that regard could not be expected until the system of payment for medical services was altered; there must be some such scheme as that foreshadowed under medical assurance. One of the beauties of the coming legislation was that hospital abuse must cease from that standpoint. Apart from that, there was only one consideration which should weigh, and that was, whether a particular case, having regard to the circumstances outside and in the hospital, could best be treated in a hospital. The only person who could decide that was the doctor, and he should be the person to sign the order for admission to the hospital. Under some such scheme as he had mentioned the question of whether the physicians and surgeons were paid and who paid them would have disappeared. The scheme of payment of the medical staffs required alteration in many respects. In most cases those gentlemen were not paid at all, and it was rumoured that in certain London hospitals they even had to pay for the privilege of being at the hospital. The idea was that they were called in to cases on the strength of their connection with a large hospital. That was an abominable system; members of the staff should be paid for their services; every skilled man, whether in or outside a hospital, should be paid at a reasonable rate for his services. It was practically impossible for a man who had not a considerable private income to become a consulting physician or surgeon at all. By the time he had qualified his parents had probably paid £1,000 for his education, and then there was further expense for rooms; and it would be about five years before he was making anything like an income. That narrowed the range of the men whom one called into consulting practice. What should decide a man being put on the staff was ability, and nothing else, and he should have a salary from the beginning; he did a great work for the hospital. There was, he believed, evidence that they were much nearer to the wished-for goal than many thought.

Sir Henry Burdett's Views.

Sir Henry Burdett, K.C.B., said it was a great disappointment to him not to have heard Professor Moore's paper, but the lines of the opener's argument were known from the full synopsis published in the Journal. He agreed with those lines of argument to the effect that it was bad for the hospitals, and worse for the profession, that the present system of unpaid eleemosynary service should be continued. He was certain that, as a mere economical factor, it would be a saving for the hospitals, taking the London hospitals as a whole, to pay the medical staff £60,000 a year rather than to continue the present system. He had stated that many years ago, and the more he looked into the question, and the more he considered it, the more convinced he was that he was right.

Abuses in the Out- and In-Patient Departments.

He would not traverse the points of Professor Moore, but in order to crystallise the matter he would relate a conversation which he had with a gentleman who was actively connected with the management of a large London hospital, to which a medical school was attached. That gentleman attacked him (Sir Henry) by saying that he must know the existing abuses in both the out-patient and in-patient departments of the hospitals, and could not understand how it was that he (Sir Henry), who was perfectly independent, did not state boldly and frankly what he knew them to be. He replied to that gentleman that it was all very well, but he had never made a statement in public, knowingly, unless he had actual data to justify it. It must be understood that, although general and detailed statements might be made, and proofs could be adduced as to abuse in the out-patient and in-patient departments, he had not got in a crystallised form a statement which he could use because it was not on authority. His informant then said he would give him two instances, and did so. Sir Henry informed him we would use them at that meeting, and the gentleman said he would be present, but he did not see him there. The gentleman said that in a clinical hospital, especially one to which a medical school was attached, the necessity and demand for material was so great that the selection of cases eliminated the social factor altogether—*i.e.* the social position and the means of the patient. Indeed, he went so far as to say that in his own out-patient department it was notorious that patients who could pay three guineas were treated, because of their value for teaching purposes, to the exclusion of poorer patients. Of course, it was known to many that there were serious abuses in regard to the admission of in-patients in the Metropolitan hospitals. The system worked out in the following way: A number of patients were set aside each day as eligible for admission, having regard to their medical and surgical requirements. Those patients were divided into two categories: those who brought cards from practitioners outside to the member of the staff whose day it was to see patients, and, secondly, those who represented the patients residing in the districts in which the hospital stood and who were physically in a condition to warrant their admission. It happened at some hospitals, and probably at most, that the first of these classes, those bringing practitioners' cards, were first considered and admitted to the available beds. But it was on record that day after day the poorer people in the district whose social needs ought to secure their admission first, especially as they came from the district supplying the funds for the upkeep of the hospital, were set aside and their cases deferred to another day because there were no vacant beds. These were grave abuses, and there were others which the gentleman concerned had confirmed. They were matters which ought not to be and could not go on.

The Right to Live.

A condition of affairs in regard to the hospitals now existed which practically meant that if the voluntary hospital was to be continually extended, and if free medical relief was to keep on growing and ramifying at the present rate, it would be impossible for the average member of the medical profession to live. Such a state of affairs was wholly wrong and was unnecessary, and it had grown up because the demand for medical treatment, as the efficiency of hospitals increased, had extended to all classes of the community. He was old enough to have had charge of a hospital forty-two years ago, in the days when hospitals were not popular, when the desire of people who fell ill was not to go to hospitals, but to keep out of them. It would be conceded that there was often good justification for that attitude, as recorded in the surgical results, as in London hospitals, even within twenty-five years, the mortality was 33 per cent. of operations where the procedure was a major one. To-day death was practically eliminated, and the mortality had fallen so low that everybody now wished to be operated upon. In fact, as far as he could make out, the general trend of the public was towards a stage revelled in by the Red Indian, when it was thought that a man did not fulfil his mission unless he were tattooed, the difference being that the Red Indian used an oyster shell, whereas we had the surgeon's knife. Mothers with sons had the idea that it was good to have the appendix removed as soon as possible, otherwise the child's future might be imperilled by his liability to appendicitis. That was an attitude which was highly complimentary to the profession, though at the same time it was condemnatory of the common sense of the people. The present was a very important opportunity for grasping the whole question of medical relief and placing it once for all on a proper basis. That basis was to secure the co-operation of all who were responsible for medical relief in every form and to put it finally on a business and practical foundation. That was quite easy of attainment in view of the report of the Poor-Law Commission, for he could see, as he stated in detail in a paper on the future of hospitals, that if they took advantage of the Poor Law and the intention of the Government to introduce medical insurance and a national insurance scheme they would get into a stage in which there would be a removal of the present abuses and so secure the right of each member of the medical profession to live. That right was now being lost, and it must be restored.

### The Classification and Grading of Hospital Patients.

There must be in every community a census of all the medical men and nurses, together with a return of all hospitals, Poor-Law infirmaries, dispensaries, and every agency for the supply of medical service in every form, including a sickness return and the medical requirements of the community. The next step was to regulate the admissions on the example of American and other foreign hospitals—namely, classifying the patients and dividing the hospitals into pavilions, having in these pavilions accommodation for every class of the community needing hospital care. Those pavilions would consist of four or five grades. There would be, first, the pavilions providing for two kinds of cases in voluntary hospitals : (1) the cases for which those hospitals were appointed—namely, people who could maintain themselves when in health, but could not meet the charge for medical service when they were ill. Next (2) a certain number of what were known as Poor-Law cases, possibly severe cases, which would be paid for by the municipality or the State. In another pavilion would be patients who desired to pay something, but not the whole cost of the treatment, because they could not afford it. A third grade of cases would be those who would pay an inclusive fee, but not the cost of the medical service. The fourth class would be those who paid everything, and who would be able in that way to refund the fees of the medical men and the cost of their treatment. In his judgment, there should also be a final class who, when wanting hospital care, could pay for it at a remunerative rate. In that way one would get rid once for all of the abuses of the so-called nursing homes.

### A Reconstituted Medical Service.

The idea meant that they must utilise all the existing Poor-Law system, all the existing voluntary system, and all the nursing and other agencies for the relief of the sick. It would mean that every patient would come to a hospital on a basis which would retain his self-respect, for if there was one thing in this country which the people wanted it was increased self-respect and greater discipline, and there was no better discipline for a young man than providing for himself if he wished to live a healthy and wholesome life. These results would be attained if the Government and the authors of the Poor-Law reports and the authorities of the medical institutions could be brought together into one representative body through a general council to whom the whole matter should be referred, and where it could be thrashed out. It meant in practice that the bringing together by co-operation of all the means of medical relief must secure the minimum of expenditure and the maximum of economy, it would enable them to utilise all the buildings at disposal and would save the State the immense cost that would be entailed by the proposal of the last speaker, if it were carried out to the abolition of the voluntary system. This system of co-operation would enable the State or the municipality to contribute the money necessary to provide the pavilions he had spoken of. The whole of the medical service would then be paid for and hospital abuse would become an impossibility.

He hoped that, from the summary he had given, made brief by the necessities of time, it would be clear what he had in mind. As one who had given much of his life, his leisure, and his means to the hospital question, he was confident that if the spirit of compromise, which was the strength of the nation and kept us where we stood, were wisely brought to bear in the settlement of this whole question, they would be much nearer a wise, complete, and absolutely satisfactory solution than had been dreamt of before in the history of the country. A solution on the lines indicated would mean that England, which possessed the best system of hospitals in the world under the voluntary system, would have under its reconstituted medical service the most perfect and the most satisfactory medical service which the world had ever seen.

### A Plea for a Paid Staff.

Dr. Heron said that as he believed practically every word which had so far been uttered, it was not necessary to enlarge very much on the subject. He once heard Mr. Sydney Holland, of the London Hospital, make a statement publicly which greatly surprised him, namely, that he was tired of hearing of the philanthropic work of the doctors in hospitals, because their reward was sufficient, in that by their work there they got into practice, got themselves recognised as being authorities on this or that subject in medicine or in surgery, and that that was a sufficient and ample reward for the work they did in hospitals. When he heard that statement he thought it was one of the most disgraceful that a man had ever made in public. It should not have been made, because no man knew better than did Mr. Holland that there were many men who had devoted their lives to hospital work who had never reached the reward which he insinuated it was the object of all to get. He was astonished that that remark had not called forth from the profession a more distinct and angry repudiation than it met with. What was a hospital to-day? He thought it fair to say that it was an institution into which anyone might go and there receive, free of cost to himself, medical or surgical treatment, provided the applicant for such treatment was not detected, while in the hospital, as being a person too well off to pay for those professional services. He thought that was not straining in any way the present position of hospitals with regard to their great work in great cities or even in small towns. It had often occurred to him that it would be well and serviceable to the public if some hospital which really believed in getting rid of hospital abuse would test how the law regarded an individual well able to pay for professional services who used that hospital while concealing the fact that he was well off, for it meant obtaining the treatment under false pretences. The law of England must be curiously framed if it could not lay hold of an impudent fraud of that kind.

Yet it was a fraud perpetrated in every large city. (Sir Henry Burdett: The Royal Free Hospital tried a case a fortnight ago, and it was dismissed.) He was not aware of that case, but thought it should have proceeded to an appeal. Hospital abuse was of two kinds.

### More About Hospital Abuse.

There was in the first place the abuse which consisted in the sweating of the medical staff, for men were not remunerated for the work they did. That should be stopped, as it was admittedly wrong. In that matter the position was the same as sixty years ago, and it would be so just as long as it was allowed to. The abuse being discussed now was patients receiving hospital treatment who were not entitled to it. The King's Fund had diminished this by appointing almoners or inspection officers. As an official of the King's Fund himself, he had come to the conclusion that although many almoners put their heart into their work, there was no doubt that to ask one man or one woman to go through the vast number of patients who attended from day to day a large general hospital, or even some of the larger special hospitals, was to ask an impossibility. The inspection which went on in many hospitals was of the most trivial and inefficient kind. And there was no official in the hospital less suited for the purpose of inspection of patients, with the object of keeping out those who ought not to be there, than the secretary. It was putting him into a false position. If he did it well the governors would be dissatisfied because the number of patients would be diminished, whereas if he did it ill he would be reported as a person who was fussy and interfering. Inspectors should be those whose whole time was devoted to the work and who were well paid for it. The remedy for the present abuse was for the best heads to get together with a view to utilising all that there was available in the shape of hospital accommodation. With the necessary safeguard the hospitals should be open to every man, woman, and child in the land, whether rich or poor, and not as a favour, but as a right, because they were citizens of the country, and when it was at all necessary the best of treatment should be given for no payment of any kind; and that where the man could pay, he should be taxed according to his means for what was done for him. That was not an Utopia, but a scheme which could be efficiently done by the medical men in this country.

### Hospital Reform and the General Practitioner.

Dr. Mackintosh remarked that hospital administration differed in various parts of the kingdom, and it was necessary to consider how the administration was conducted in those centres in order to arrive at any conclusion. He agreed that the system of hospital administration must be unified. The medical profession must be fairly represented and adequately remunerated and the public were to receive their just and fair treatment. The opener mentioned three systems of hospital administration: voluntary, municipal, and Poor Law. In regard to remuneration, there were different sections of the profession to be considered: the hospital physician and surgeon, the hospital administrator, the resident physician, the general practitioner outside. Municipal hospitals had dealt with fever for some years, and now practically everybody sent a child which had fever to a fever hospital. One should consider what that meant for the general practitioner, for twenty years ago the medical practitioners treated those cases themselves. He would therefore deprecate any sudden move in reform because the medical practitioner must be considered. Moreover, sanatoria were springing up all round. He agreed with the payments of the staffs of voluntary hospitals; in fact, every man should be paid for what he did efficiently. The advantage derived from the holding of the position of senior physician or surgeon to a hospital might be considered indirect payment, but it was not by any means adequate. In some hospitals an honorarium was given to the senior staff, and they received practically all the students' fees. He would be in favour of payment on another scale; the officer should be paid at a rate commensurate with the services rendered. The assistant surgeons and assistant physicians did a great deal of work, but were not paid, and they did not receive the students' fees, nor any kudos, unless it were reflected. If attention were not paid to the assistant surgeons and physicians, where were the consultants to come from? It was urgent that the matter should be looked into. Sir Henry Burdett said it was necessary to have a voluntary hospital in connection with a teaching school because of the need for material. One knew that teaching could not be done without material, but he did not follow the argument that a hospital should exist simply for the supply of clinical material; it existed for the care and cure of the sick, and in University towns there was the added function of teaching young medical men. It was no justification to establish a hospital because a school was there, the school was established because the hospital was there. There were always a number of cases suitable for hospital; where this was not so a hospital should not exist. From a twenty-four years' experience he could say that the amount of abuse inside hospitals in Scotland was very slight. There were people in all the hospitals sent to have major operations performed, wives of the industrial class who could not afford to have the operation done privately, and the only claim for admission to the hospital was that the medical man attending the patient knew the needs and circumstances surrounding her, and recommended the patient. Much could be said both for and against recommendation cards. A hospital had to be maintained, and unless funds were forthcoming it could not be kept up. There were occasions when recommendations might be useful, but they should not be used as passports for treatment. The only grounds on which a patient could be admitted for treatment at a hospital were the surgical and medical, that the case was suitable for hospital treatment.

### Special Hospitals.

Mr. Richard Kershaw (Central London Throat and Ear Hospital) said the feeling he had with regard to medical relief was that it should be as free as the air we breathed. He thought the co-ordination of medical relief was bringing the reform required quicker than anything else, as there was a fair prospect of the Government taking over the hospitals of the country. The health of the individual should be the concern of the State. He was entitled to speak on the subject of hospital abuse, because it was closely allied to his work and he had spent over thirty years of daily work to try and obviate such abuse. When he changed from a general to a special hospital he saw a great difference in the social position of the patients attending. For many years he wrote a letter to everyone who recommended a patient, unless the patient were obviously of the hospital class, asking if it was the wish of the person that the patient should continue attending, and, if an operation were wanted, whether he would care to be present at it. But it was only rarely that such letters met with any encouragement. The patients numbered four thousand in the first year he went, and now they numbered eleven thousand per annum. Thirty-nine per cent. of the out-patients and 74 per cent. of the in-patients were last year sent direct by medical practitioners. Of the remaining 60 per cent. of out-patients probably 30 per cent. were obviously suitable, but perhaps 15 per cent. or 20 per cent. should not be there. Of those he asked the name of their doctor, and he had written such doctors consistently asking whether they desired the patient to come again. But he did not think he received 10 per cent. of replies. The remedy was in the hands of the practitioners. The special hospitals were doing a work which was just outside the work of the practitioner, who could not be expected to have the necessary special armamentarium. Some of the cases, though not at the time serious, would become so if left untreated.

### The Ticket System.

Dr. Saundby (Birmingham) said the modes of admission to hospitals had been much discussed, and he thought one of the reasons of lack of agreement was that hospitals were not worked the same all over the country. The old ticket system had been strongly condemned many times by the British Medical Association and resolutions had been passed, and yet it had many advantages; it got rid of difficulties which any open system presented. At his hospital in Birmingham a ticket system was worked in such a modified way that it did only good. Every subscriber was given a number of tickets, both in-patient and out-patient, and no patient was allowed to come to the out-patient department without a ticket. Still, anybody applying for relief there was relieved as a casualty case. It enabled the out-patient work to be limited, and it was there one found an overwhelming mass of trumpery cases. Telling a patient that he must bring a ticket when he came again reduced the attendance. It was better than the system introduced some years ago at Queen's Hospital, where tickets were replaced by a shilling registration fee. That resulted in a loss of subscribers which was never recovered. It was often found that poor patients had to beg the shilling. In some cases the fee was extended until as much as 5s. was paid for a fortnight's treatment, and that led to a distinct scandal, for there were special hospitals in London which advertised regularly in the cheap Press which was read by poor people, stating the amount of the registration fee and the kind of diseases which the hospital undertook to treat. Those advertisements could not be said to aim at increased subscriptions, for they did not go among that class of people; they were more likely to get patients.

### Recommended Patients.

The British Medical Association should take that question up. A case was before the British Medical Council at its last sitting in which the man defended himself on the ground that he was doing no more than the small special hospitals were doing. And there was some force in the defence. He wished the idea that recommendations from practitioners should be the means of getting into hospitals was a practical rule, but Mr. Kershaw had shown that it was difficult to get doctors to express an opinion. Attention was given to any recommendation from a doctor; cases sent by them were taken in without question, and often the result was scandalous abuse, several instances of which he detailed. One patient was said to have hæmorrhage from the stomach, and of course he was taken in. On investigation it was found that the hæmorrhage occurred three weeks before. He thought the medical profession could not be trusted to decide the right of entry to hospital. He did not follow the statement that every man, woman, and child in the country should have the right of going to a hospital. Surely the only claim was their medical or surgical need. Sir Henry Burdett had formulated a scheme which seemed feasible, but it did not yet exist, and until then he thought they would be content to go on as at present. (Sir Henry Burdett assured Dr. Saundby that he had spent the ten weeks between his speech on the subject and the present time in seeing those interested, and he believed the solution was nearer than was thought.) It was easy to manage the nursing-home side of the question. He had felt that the matter was so serious that he had shrunk from having anything to say about it.

### The G.P.'s View.

Dr. Hugh Ker spoke from a general practitioner's point of view, and he could speak of the hard conditions of practice near a large out-patient department. It even affected Friendly Societies, who used to employ medical practitioners to attend their members. He heard of a case in which a large Friendly Society in the neighbourhood of Guy's Hospital had discontinued the appointment of a medical man for the reason that there was no difficulty in getting medical attention at the out-patient department. At the Bolingbroke Hospital, in South London, a system was adopted under which no out-patient would be seen without a letter from a practitioner; that department was entirely consultative, as recommended by the British Medical Association. The secretary of that hospital said it had not only resulted in little abuse, but the teaching material had been very good. During 1908 26,450 cases attended the out-patient department. He thought that was the true system to go upon. If it could be made a success in a small hospital of sixty beds it could be done on a larger scale. Unless some system were adopted which enabled the medical man to make a living, a large number of practitioners must go to the wall.

### The Economic Aspect.

Dr. Buist said the discussion had ranged over a wide interpretation of economics. He did not accept the suggestion that the staff were justly paid from the fees of the students. The question should be looked at from a national as well as from an individual point of view. It was beneficial to the individual student that he should be educated, but it was better for the country that he should be well educated and a profession supplied to him. The burden should rest on the nation, not on the student. It was not a question of there being less total work in the profession, but a different distribution among its members. The economic side was really a national one, and the charitable aspect of hospitals must gradually be curtailed. Whatever view was taken of the social construction, those present could not but regard the proposal for the co-ordination of the whole hospital system of the country with great favour.

### Do not Nationalise.

Dr. Greenwood said the charitable spirit in the past was no doubt largely influenced by the teaching of the Church. The charitable feeling was not one to be discouraged, and he said that because during the present age there had been a feeling against it. With regard to deferring to public views, it must be remembered that they varied very much, and he thought some public movements at the present day should be resisted. With regard to the municipalisation of hospitals, at present the burdens on the public were very great, and would be greater in the future, and he did not think the avenues of contribution to the hospitals by the rich should be closed. He did not think any good would ensue to either the profession or the public by the municipalisation of hospitals; but he agreed with co-ordination. He did not think that in every London hospital the members of the staff should receive salaries; a certain amount of work the staffs might be able to do for nothing. He did not think the profession would stand in a better light in the eyes of the public if they demanded payment for everything. General practitioners suffered much by the readiness with which people could get hospital treatment, and many members of the community could get practically hospital treatment in their own homes. Some hospitals had departments for the reception of private patients, and there was no objection to that; but general practitioners must resist the tendency to drag them into a nationalisation of medical service.

### Compromise.

Dr. Luckham claimed to have some grounds for speaking on the question, as he had been successively honorary visiting member of a staff of an honorary hospital, a doctor in dispensary work, club work, had been attached to a Poor Law infirmary, and had done Poor Law work outside. Many would agree that the charitable work done by a doctor was a greater pleasure and was better done than the ill-paid work of dispensaries and clubs, and therefore one must consider whether the work was being improved by doing away with the charitable part of it. That would not be so unless the work were properly paid for; one was apt to scamp ill-paid work. Compromise must be carried out with regard to the admission of patients; the doctor could not be depended upon to be the best judge of the cases to send to hospital. The subscribers' letter should be endorsed by the medical man, but that should afterwards be scrutinised by the hospital authorities. In conclusion he expressed his anxiety for a wider recognition of the gratuitous work done by members of the medical profession.

### Hospitals and the Rates.

Professor Morrison said that Professor Moore and Sir Henry Burdett appeared to foresee a complete revolution in hospital administration and medical service generally in the country. The vista which those speakers opened up appealed to him, for it seemed to be scientific and businesslike, and to provide a solution of the great problem connected with hospitals. He would acclaim the day foreshadowed by Sir Henry Burdett if he should live to see it. With regard to payment of staffs and hospitals, a moral obligation rested on any community to maintain its indigent and sick. If that proposition were granted, the provision should be complete. The idea that members of hospital staffs were sufficiently compensated by what was called kudos, or that they were sufficiently compensated by the financial return which many of them received, was beside the question. The members of the profession with princely incomes were very few, and one had to consider the general run, particularly the junior members, who had to go on for years before they could make a good income. There might be some justice in the remark that if the public hospitals were municipalised there would be a considerable diversion of charitable income. But if hospitals were put on the rates, the contribution per head would be much smaller than that paid by the ordinary annual subscriber. The question of the enormous number of people now attending hospitals should receive more attention than it had hitherto. Last year alone, in the town in which he lived, with a population of half a million, there were 200,000 out-patients at the public hospital. Out of those there must have been an enormous number who were able to pay for medical service. If hospitals became municipalised, it did not necessarily follow that the general practitioner would be starved out; it was possible to combine with hospitals a satisfactory system of provident dispensaries.

### Consultative Hospitals.

Dr. Lauriston Shaw said there was no necessary charitable basis of a hospital, and if one could imagine a society in which the great virtue of charity was entirely lost, and everybody was merely self-seeking and looking after his own physical advantage, there would be hospitals. However wealthy people might be, it was an advantage to them, under certain circumstances, to be treated in an institution like a hospital; it was only there that the highest professional object could be attained, where the structures were properly made, and there was an organisation for constant attendance and nursing of the highest quality. For many people it was a question not only of efficiency, but also of economy. Imagining a State without charity which had developed hospitals on those lines, what would they do when the great spirit of charity suddenly came upon them and they recognised that the things they had been making for themselves must now be provided for others who were not able to use them? He could not imagine that they would make such a hash of it as our present system of hospitals was in. They would not destroy the special functions of a hospital by mixing it up with some unnecessary function. Charity should be always in advance of the general community; charitable effort always was. As soon as the community recognised what a few advanced thinkers had recognised as necessary for the suffering, the need for charity disappeared. That gave a clue as to the best relationship between charity and medical service. The community recognised that for ordinary ailments every member of the community must be provided for. But charitable people should be induced to get ahead of the community, and provide by charity also the extraordinary requirements. Then the general practitioner could be left to deal with the ordinary requirements and leave the specialist to deal with the more complicated requirements of the sick poor. If one could make hospital treatment, the treatment not for everybody who required anything, but for those who required the more advanced treatment, the profession would not be divided into two camps—the member of the hospital staff, who was constantly interfering with the economic position, and the general practitioner. He saw no difficulty in establishing the fact that no patient should receive hospital treatment on charity at an institution unless arrangements had been made by some other authority to deal with his simpler ailments. Charitable hospitals should not be for the treatment of a patient merely because he was poor, but for the treatment of those whose cases demanded some special form of medical service, either because a specialist was required or because it needed prolonged study.

The meeting then concluded, without a reply, as Professor Moore had left.

